# Unveiling the mechanism of dye-decolorizing peroxidase (DyP): unique anthraquinone-directed catalysis exposes a dual-function peroxidase

**DOI:** 10.1128/aem.00282-26

**Published:** 2026-04-22

**Authors:** Peilin Lin, Feng Tian, Hao Chen, Yongming Lu, Liuqing Li, Rong Jia

**Affiliations:** 1 School of Life Sciences and Medical Engineering, Anhui University 12487, Hefei, Anhui Province, China; 2 Anhui Key Laboratory of Modern Biomanufacturing, Anhui University 12487, Hefei, Anhui Province, China; 3 Institute of Health and Medicine, Hefei Comprehensive National Science Center 604255 , Hefei, Anhui Province, China; Universidad de los Andes, Bogotá, Colombia

**Keywords:** dye-decolorizing peroxidases, anthraquinone dyes, catalytic mechanism, transformation pathway

## Abstract

Dye-decolorizing peroxidases (DyPs) are heme-containing peroxidases that catalyze the reduction of H_2_O_2_ to water, accompanied by the oxidation of diverse substrates, including lignin-related phenolic and non-phenolic compounds, as well as a wide range of synthetic dyes. These enzymes exhibit a marked preference for decolorizing recalcitrant anthraquinone dyes, setting them apart from other peroxidases; however, the molecular mechanism underlying this preference remains to be elucidated. This study explores the catalytic profile of recombinant *Il*-DyP4, a dye-decolorizing peroxidase from *Irpex lacteus* F17, to clarify the molecular basis of its substrate preference. We found that *Il*-DyP4 exhibits remarkable H_2_O_2_ tolerance in the oxidation of anthraquinone dyes, maintaining approximately 50% activity even at H_2_O_2_ concentrations above 20 mM, while its activity toward phenolic substrates decreases by 50% at only 0.6 mM H_2_O_2_ concentration. These results demonstrate the distinct capability of *Il*-DyP4 to catalyze anthraquinone dyes at high H_2_O_2_. Analysis of the intermediates of Reactive Blue 19 transformation showed that this catalytic ability is linked to the bifunctional activity of *Il*-DyP4: the enzyme couples peroxidative oxidation with hydrolytic cleavage to rapidly scavenge H_2_O_2_, thereby averting self-inactivation of the enzyme. In contrast, guaiacol oxidation by *Il*-DyP4 followed the canonical peroxidase route, proceeding exclusively via C–C radical coupling to yield oligomeric products. These findings provide a mechanistic rationale for the intrinsic preference of DyPs for anthraquinone-based dyes and highlight *Il*-DyP4 as a resilient, high-performance biocatalyst for remediating dye-contaminated wastewater.

**IMPORTANCE:**

Here, we reveal that the long-sought specificity of dye-decolorizing peroxidase (DyP) for anthraquinone dyes arises from a bifunctional mechanism orchestrated by *Il*-DyP4, encompassing both peroxidase-mediated oxidation and hydrolytic cleavage. Elucidating this dual chemistry not only redefines the catalytic behavior of the DyP family but also provides a mechanistic cornerstone for engineering robust biocatalysts to eliminate recalcitrant dye pollutants in industrial wastewater.

## INTRODUCTION

Dye-decolorizing peroxidases (DyPs, EC 1.11.1.19) are a class of heme-containing peroxidases that utilize non-covalently bound protoporphyrin IX (heme) as a cofactor. They belong to the largest superfamily of heme peroxidases widely distributed across bacteria, fungi, archaea, and Metazoa (1, 2). Based on sequence homology, DyPs are categorized into four subfamilies (A–D) (3, 4). Subfamilies A, B, and C are primarily bacterial in origin, while class D DyPs are exclusively found in fungi (5). The sequence identity among these four classes is generally low: class D DyPs share only 7%, 7%, and 16% identity with classes A, B, and C, respectively (6). Later, Yoshida and Sugano (3) constructed a phylogenetic tree from all DyP sequences available in the InterPro database (25,475 entries as of 17 February 2022) and classified them into three groups: P (class B DyPs), I (class A DyPs), and V (classes C and D DyPs). Class P is the shortest class, while Class V is the longest, containing approximately 300 and 500 amino acid residues, respectively.

The first DyP was isolated from the basidiomycete *Bjerkandera adusta* (initially misidentified as *Geotrichum candidum* Dec 1) and was characterized as a glycosylated heme protein (containing ~17% sugars), with a pI of 3.8 and a molecular mass of 60 kDa (7). Crystal structure analyses have revealed that DyPs possess a unique tertiary structure composed of both α-helices and β-sheets forming a ferredoxin-like fold, which distinguishes them from classical lignin-degrading enzymes such as manganese peroxidases (MnPs, EC 1.11.1.13), lignin peroxidases (LiPs, EC 1.11.1.14), and versatile peroxidases (VPs, EC 1.11.1.16) (1, 8–10). In DyPs, the heme is anchored to the C-terminal domain, and a conserved histidine in the adjacent proximal pocket serves as the axial ligand to the iron, as is typical for most heme peroxidases (11–13). Uniquely among heme peroxidases, DyPs possess a conserved acidic residue (usually aspartic acid) in the distal heme pocket (14). Together with a nearby arginine on the distal plane of the heme, it is believed to facilitate O–O bond cleavage in H_2_O_2_ and to promote proton transfer (15).

Despite these structural differences, DyPs share a similar catalytic cycle with other classical peroxidases. The catalytic cycle is initiated by the reaction of the resting enzyme with (H_2_O_2_), forming a Fe(III)–OOH intermediate (Compound 0), which undergoes heterolytic O–O bond cleavage to yield Compound I (Cpd I, Fe(IV)=O Por•^+^) and water (16). Cpd I of class B DyPs is relatively stable and reacts slowly with reducing substrates, making it amenable to detection via UV–vis spectrophotometry and electron paramagnetic resonance (EPR) spectroscopy (17–20). This intermediate can undergo either a one-electron reduction to form Compound II (Cpd II, Fe(IV)–OH Por) or a two-electron reduction to regenerate the resting state (21, 22). In contrast, Cpd I, formed by class C and D DyPs, is short-lived, as the porphyrin radical rapidly migrates to a nearby redox-active amino acid (usually Trp or Tyr), forming Compound I* [Cpd I*, Fe(IV)–OH Por•…aa] containing an amino acid radical (23). This radical migration is closely associated with substrate oxidation occurring at the enzyme surface (24, 25). Although H_2_O_2_ is essential for the peroxidase catalytic cycle, excess H_2_O_2_ can irreversibly convert Cpd II into Compound III (Cpd III) (26, 27), leading to enzyme inactivation (28, 29).

DyPs have been demonstrated to oxidize a broad spectrum of substrates, including typical peroxidase substrates such as phenols, 2,2′-azino-bis(3-ethylbenzothiazoline-6-sulfonic acid) (ABTS), Mn²^+^, and lignin model compounds. More notably, DyPs can decolorize various synthetic dyes, including anthraquinone, azo, phenazine, and triphenylmethane dyes (4, 30–32). Interestingly, DyP from *B. adusta* exhibits a higher decolorization rate for anthraquinone dyes compared to azo dyes, indicating that its substrate specificity differs from those of the other heme peroxidases, such as LiP and MnP (7). Yoshida and Sugano (6) summarized that DyP exhibits the highest activity toward anthraquinone dyes, with a highly acidic optimal pH of 3.2. Linde et al. (25) suggested that long-range electron transfer (LRET) between the heme cofactor and the radical-forming exposed tryptophan (Trp-377) was suggested as a potential mechanism for the oxidation of anthraquinone-type RB19 (Reactive Blue 19) by *Auricularia auricula-judae* DyP. Nevertheless, the molecular basis for the preference of DyPs toward anthraquinone dyes is not well understood.

Anthraquinone dyes are particularly resistant to transformation due to their high redox potential (5, 33). Conventional lignin-degrading peroxidases (MnPs, LiPs, and VPs) are more effective at decolorizing azo dyes and typically show limited activity toward anthraquinones (34–37). Structurally, anthraquinone dyes are composed of three benzene rings with the central ring bearing two carbonyl groups, forming a highly conjugated π-electron system. This structure imparts high thermodynamic stability and antioxidant properties (33, 38). As a result, anthraquinone dyes are widely used in textiles, pharmaceuticals, and fuels. With increasing industrial demand, they have become the second-largest class of synthetic dyes after azo dyes, contributing significantly to dye-contaminated wastewater (5). In addition to synthetic dyes, phenolic compounds usually serve as peroxidase substrates. These aromatic compounds contain a hydroxyl group (–OH) directly attached to a benzene ring, making them prone to oxidation and leading to the formation of quinonoid intermediates. Phenolic compounds are prevalent in many industrial sectors (39, 40). Similar to anthraquinones, these compounds are highly recalcitrant and resistant to degradation via conventional chemical, photolytic, or biological processes.

DyPs have demonstrated considerable potential for the remediation of dye-contaminated wastewater. Nevertheless, the mechanistic basis for their preferential activity toward anthraquinone dyes remains poorly understood, and the transformation pathways of anthraquinones mediated by DyPs have yet to be fully elucidated. This study seeks to address these gaps by examining the catalytic behavior of a recombinant fungal DyP (*Il*-DyP4) toward anthraquinone dyes in direct comparison with phenolic substrates. Our findings support a bifunctional transformation pathway that combines peroxidase-like oxidation with hydrolytic steps, facilitating the preferential degradation of anthraquinone dyes. This work not only underscores the functional versatility of DyPs but also enhances our comprehensive understanding of their catalytic characteristics.

## MATERIALS AND METHODS

### Expression and purification of recombinant *Il*-DyP4

As we previously determined crystallographically (*Il*-DyP4 residues 6–450, PDB ID: 7D8M) (19), the enzyme binds a single heme cofactor and has a calculated molecular mass of ~54 kDa. The pET28a-*Il*-DyP4 expression vector carrying the *Il*-DyP4 gene (GenBank: MG209114) and the corresponding *Escherichia coli* Rosetta (DE3) expression and purification protocols were both established by Duan et al. (31). In short, the recombinant *E. coli* was cultured in LB medium supplemented with 20 μg/mL kanamycin and induced with 0.5 mM isopropyl-β-D-thiogalactoside at 37 °C for *Il*-DyP4 overexpression. After induction, the cells were harvested by centrifugation at 4,500 × *g* and resuspended in 50 mM Tris-HCl buffer (pH 7.5) containing 0.02 mM phenylmethylsulfonyl fluoride and 4 mM ethylenediaminetetraacetic acid (EDTA). Inclusion bodies were released by ultrasonication, denatured in 50 mM Tris-HCl (pH 7.5) containing 8 M urea and 0.5 mM EDTA for 3 h, and renatured at 4°C for 36 h in 10 mM sodium acetate buffer (pH 6.0) containing 0.8 M urea, 1 mM EDTA, and 5 μM hemin. Excess hemin was removed by dialysis in sodium acetate buffer (pH 6.0) at 4°C. Insoluble proteins and impurities were removed by centrifugation, and the crude enzyme was purified using Ni-NTA affinity chromatography following the manufacturer’s instructions. The final protein concentration and buffer exchange into sodium acetate (pH 6.0) were performed using Amicon Ultra 10 kDa ultrafiltration tubes at 4°C. The purity and molecular mass of recombinant *Il*-DyP4 were verified by SDS-PAGE, and its UV–vis spectrum displayed the characteristic Soret band of the heme at 406 nm (Fig. S1).

### Activity assays and steady-state kinetic analysis

Three phenolic compounds and three anthraquinone dyes were used as substrates. The oxidation activity for three phenolic substrates was measured based on the molar absorbance of the corresponding reaction product of guaiacol (*ε*_465_ = 12,100 M^−1^ cm^−1^), 2,6-dimethoxyphenol (*ε*_468_ = 27,500 M^−1^ cm^−1^), and hydroquinone (*ε*_247_ = 21,028 M^−1^ cm^−1^). In the case of dyes, enzymatic activities were estimated from substrate decolorization, using the molar absorbances of Reactive Blue 19 (RB19, *ε*_595_ = 10,000 M^−1^ cm^−1^), Reactive Blue 5 (RB5, *ε*_600_ = 9,100 M^–1^ cm^–1^), and Acid Blue 25 (AB25, *ε*_600_ = 6,800 M^−1^ cm^−1^). Each 1 mL reaction contained 10 mM sodium tartrate buffer (pH 3.5), 0.1 mM dye (or 1 mM phenolic substrate), and 0.25 μg/mL *Il*-DyP4. Enzyme-free controls were run in parallel. The reaction was initiated by adding 0.1 mM H_2_O_2_ and was allowed to proceed for a 2 min incubation period at 35°C before the absorbance was measured using a UV–vis spectrophotometer (UV-2100, Unico). Then the specific activity was calculated using equation 1:


(1) $$\text{Specific activity (U/mg)}=(V\times \Delta A\times 10^{6})/(V_{1}\times c\times t\times l\times \varepsilon )$$

where *V* = total reaction volume (mL); Δ*A* = change in absorbance; *V*_1_ = volume of enzyme added (mL); *c* = enzyme concentration (mg/L); *t* = reaction time (min); *l* = cuvette path length (cm); and ε = molar extinction coefficient (M⁻¹ cm⁻¹).

For steady-state kinetic analysis, the reaction conditions were as above, except that *Il*-DyP4 concentration was 3.3 nM, and substrate concentrations ranged from 0.01 mM to 0.5 mM. The reaction rate was calculated using equation 2:


(2) $$Reaction\;rate\;(mol\cdot L^{-1}\cdot min^{-1})=\Delta A\;/\;(\varepsilon \times l\times t)$$

Michaelis–Menten kinetic parameters (*K_m_*, *k*_cat_, and *k*_cat_/*K_m_*) were obtained by fitting the data to equation 3 and 4 using nonlinear least squares regression:


(3) $$v=V_{max}\left[S\right]/(K_{m}+[S])$$

(4) $$k_{cat}=V_{max}/{\left[E\right]}_{total}$$

where *v* is the initial reaction rate, *V*_max_ is the maximum rate, [*S*] is substrate concentration, and [*E*]_total_ is the total enzyme concentration.

### EPR spectroscopy

To analyze the heme iron spin state and protein radical formation in *Il*-DyP4, EPR spectra were recorded using a Bruker EMX plus 10/12 spectrometer equipped with an Oxford ESR910 Liquid Helium cryostat (Bruker, Ettlingen, Germany) at the High Magnetic Field Laboratory, Chinese Academy of Sciences (Hefei, China). Each 250 μL sample contained 40 μM *Il*-DyP4, 0.4 mM substrate, and 10% glycerol (cryoprotectant) in 10 mM sodium tartrate buffer (pH 3.5). H_2_O_2_ was omitted to analyze the resting state and included to analyze the activated enzyme. After a 20 s incubation, spectra were collected under these measurement conditions: temperature 5 K, microwave power 2 mW, receiver gain 10,000 G, modulation amplitude 2 G, scan ranges 500–4,500 G (wide) and 3,250–3,450 G (narrow).

### LC–MS analysis of substrate transformation products

To investigate the substrate transformation pathway of *Il*-DyP4, reaction intermediates were analyzed by LC–MS (LTQ Orbitrap XL, ThermoFisher). The 15 mL reaction system contained 0.1 mM dye or 1 mM phenol substrate, 0.5 μg/mL *Il*-DyP4, 0.1 mM H_2_O_2_, and 10 mM sodium tartrate buffer (pH 3.5). After 5 min at 35°C, the reaction was quenched by rapid freezing in liquid nitrogen. Samples were freeze-dried, redissolved in 1 mL of water, and centrifuged at 20,000 × *g* for 10 min to remove insoluble material. Supernatants were analyzed via LC–MS using a TC-C18 column (Thermo Scientific, Hypersil GOLD C18, 100 × 2.1 mm, 3.0 μm). The column temperature was maintained at 25°C, and the injection volume was 20 μL. In negative ion mode, 5 mM ammonium acetate and methanol served as mobile phases in a linear gradient from 5% to 85% methanol (vol/vol) at 0.2 mL/min. In positive ion mode, 0.1% acetic acid and methanol were used across a 30%–90% gradient at 0.6 mL/min. Full scan ranges: *m*/*z* 50–800 (negative mode) and *m*/*z* 100–1,000 (positive mode).

### Molecular docking of *Il*-DyP4 with substrates

The molecular docking study has been performed using the structure of *Il*-DyP4 (PDB ID: 7D8M) by AutoDock-GPU (AutoDock 4.2.6) (41). All water molecules were removed. The protein was pre-processed by adding hydrogen atoms and assigning the protonation states of ionizable residues under neutral pH conditions. The docking grid was centered around catalytic residue W380 with grid center coordinates (*x*, *y*, *z*) = (4.12, 41.266, 12.302) Å and dimensions of 30 × 30 × 30 Å. A conformational search algorithm explored ligand flexibility using grid maps to evaluate protein–ligand interactions. The docking results were clustered, and the conformation with the lowest binding free energy was selected as the most probable binding mode. This selection was further validated to ensure it maintained key interactions and was free of steric hindrance. The resulting complexes were analyzed using LigPlus, and interaction visualizations were generated in PyMOL.

## RESULTS AND DISCUSSION

### Oxidation characteristics of *Il*-DyP4

To investigate the oxidation behavior of *Il*-DyP4 under varying H_2_O_2_ concentrations, three anthraquinone-type and three phenolic substrates were used to determine enzyme activity. For the anthraquinone substrates, the experiment established 11 H_2_O_2_ concentration gradients: 0.1, 0.4, 1.0, 4.0, 10, 20, 60, 100, 150, 200, and 250 mM. For the phenolic substrates, 10 gradients were set up: 0.05, 0.1, 0.2, 0.4, 0.6, 1.0, 2.0, 4.0, 6.0, and 10.0 mM.

**Fig 1:**
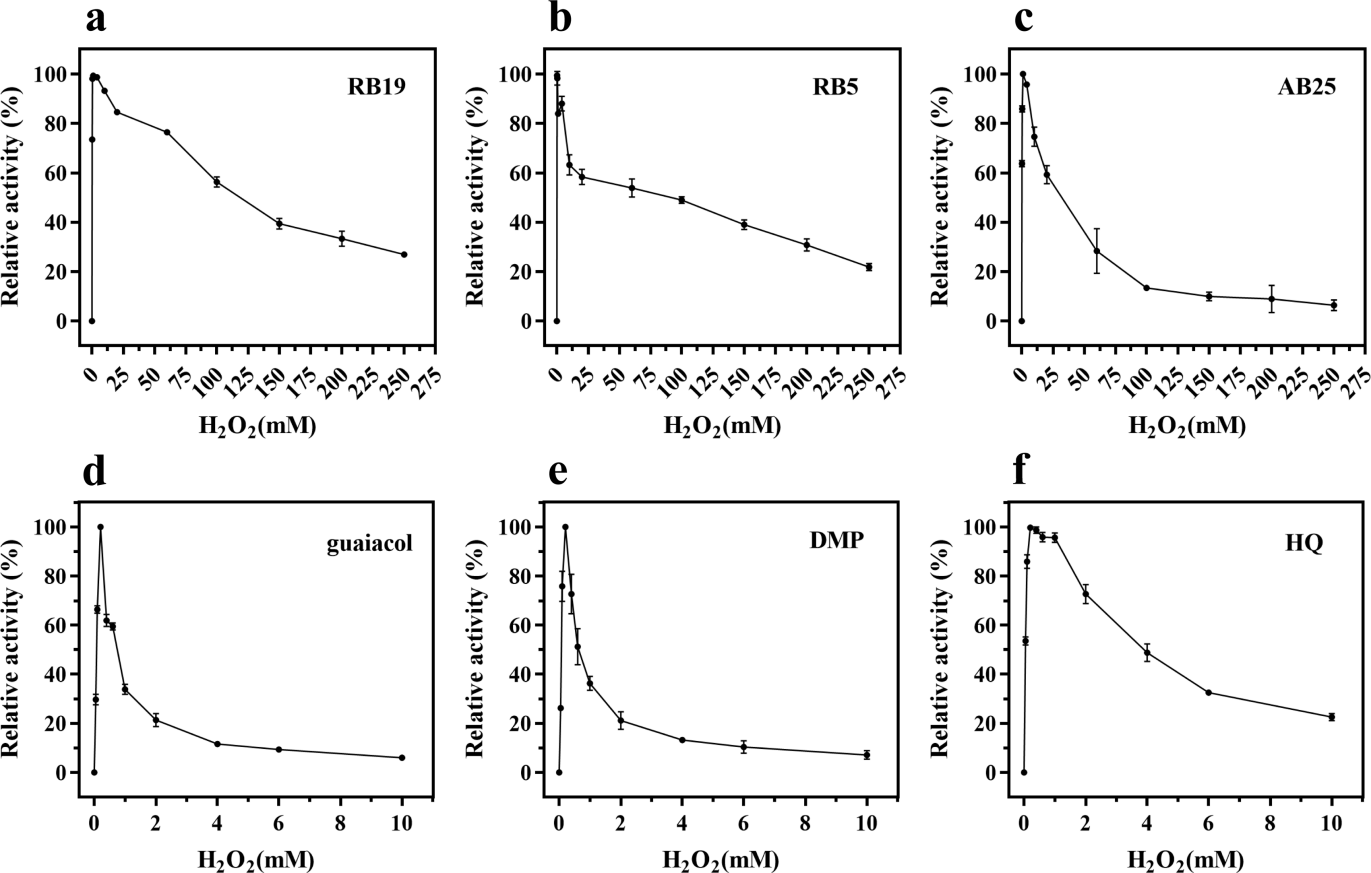
Effect of H_2_O_2_ concentration on the oxidation activity of *Il*-DyP4. The reactions were performed at 35°C for 2 min. (**a–c**) Anthraquinone dyes (RB19, RB5, and AB25) tested with H_2_O_2_ concentrations ranging from 0.1 to 250 mM. (**d–f**) Phenolic compounds (guaiacol, DMP, and HQ) tested with H_2_O_2_ concentrations ranging from 0.05 to 10 mM. Data are presented as mean ± SD (*n* = 3). The specific activities corresponding to 100% relative activity are as follows: Reactive Blue 19 (151.2 U/mg), Reactive Blue 5 (75.6 U/mg), Acid Blue 25 (135.0 U/mg), guaiacol (182.2 U/mg), 2,6-DMP (15.3 U/mg), and hydroquinone (227.7 U/mg).

As shown in Fig. 1, the ability of the anthraquinone substrates to protect *Il*-DyP4 from deactivation was significantly superior to that of phenolic compounds under high H_2_O_2_ concentrations. Specifically, the optimal H_2_O_2_ concentration for anthraquinone dyes (RB19, RB5, and AB25) was approximately 1.0 mM (Fig. 1a through c). The enzyme retained significant stability beyond this peak, maintaining 50%–60% relative activity at 20 mM H_2_O_2_, with measurable activity persisting up to 250 mM. For example, RB19 and RB5 oxidation retained ~34% and ~31% activity at 200 mM H_2_O_2_, respectively. Conversely, the optimal concentration for phenolic substrates (guaiacol, DMP, and HQ) was fivefold lower (~0.2 mM). Inactivation was rapid and severe: activity toward guaiacol and DMP fell to ~21% at 2 mM H_2_O_2_, and near-complete inactivation (<10% residual activity) occurred at 10 mM H_2_O_2_ for all tested phenolics (Fig. 1d through f). These results indicate that anthraquinone substrates enhanced the catalytic stability of *Il*-DyP4 under high H_2_O_2_ concentrations, protecting the enzyme from H_2_O_2_-induced inactivation. It is well documented that the stability of DyP-catalyzed reactions depends on the oxidant (H_2_O_2_) and available reducing compounds (substrate); when substrate is depleted, the enzyme is readily inactivated by H_2_O_2_ (42).

To further assess this effect, steady-state kinetic parameters were determined for *Il*-DyP4 oxidation of RB19 and guaiacol at varying H_2_O_2_ concentrations (0.4, 2, 20, and 50 mM). As shown in Table 1, *Il*-DyP4 maintained robust activity toward RB19 even at 50 mM H_2_O_2_ (*k*_cat_ = 509 ± 32 s⁻¹), while the activity toward guaiacol was nearly abolished, precluding the calculation of *k*_cat_. This reinforces the hypothesis that anthraquinones confer greater protection against H_2_O_2_-induced deactivation compared to phenols. The observed kinetic behavior of *Il*-DyP4 is consistent with its specific structural characteristics. *Il*-DyP4 possesses two spatially distinct substrate binding sites: the small oxidant H_2_O_2_ enters the internal heme cavity to drive the formation of the catalytic intermediate (Compound I), whereas bulky substrates bind to the protein surface around residue W380, which is approximately 11 Å away from the heme (16). Oxidation of the surface-bound substrate is mediated by the LRET pathway connecting W380 to the heme cofactor. Therefore, high concentrations of H_2_O_2_ affect the formation rate of Compound I, thereby influencing the catalytic turnover rate (*k*_cat_) of reductive substrates. For different types of substrates (such as RB19 and guaiacol), differences in their structure and properties result in variations in their catalytic rates.

**TABLE 1:** Steady-state kinetic constants for the oxidation of RB19 and guaiacol by *Il*-DyP4 under different concentrations of H_2_O_2_

Parameter	RB19 at H_2_O_2_ concn (mM) of:				Guaiacol at H_2_O_2_ concn (mM) of:			
	0.4	2	20	50	0.4	2	20	50
*k*_cat_ (s^−1^)	645 ± 64	1120 ± 8	627 ± 37	509 ± 32	103 ± 6	49 ± 9	58 ± 5	–^*a*^
*K*_*m*_ (μM)	348 ± 49	57 ± 7	130 ± 16	109 ± 14	55 ± 6	22 ± 7	27 ± 5	–
*k*_cat_/*K*_*m*_ (×10^6^ M^−1^ s^−1^)	1.9 ± 0.5	19.6 ± 0.5	4.8 ± 0.5	4.7 ± 1.0	1.9 ± 0.4	2.2 ± 1.6	2.2 ± 0.7	–

^*a*^ –, unable to calculate because enzyme activity is too low.

Additionally, kinetic constants for a broader range of substrates, RB19, RB5, DMP, and guaiacol, were measured at 0.1 mM H_2_O_2_ (Table 2). The turnover rate (*k*_cat_) for RB19 was 251 ± 30 s^−1^, more than double that of the phenolic reference guaiacol (122 ± 7 s^−1^). However, the overall catalytic efficiencies (*k*_cat_/*K*_*m*_) are comparable due to the significantly lower *K*_*m*_ of guaiacol. This kinetic behavior aligns with the structural features of fungal DyPs: small phenolic substrates like guaiacol can access the heme pocket for tighter binding (25), whereas bulky anthraquinones are restricted to surface sites, where oxidation occurs via rapid LRET (43). Indeed, Linde et al. (43) demonstrated that a surface tryptophan residue in *Auricularia auricula-judae* DyP serves as a high-turnover site for phenolic oxidation, effectively outperforming the lower-activity heme channel for specific substrates.

**TABLE 2:** Steady-state kinetic constants for the oxidation of two anthraquinone substrates and two phenolic substrates by *Il*-DyP4 using 0.1 mM H_2_O_2_

Substrate	*k*_cat_ (s^−1^)	*K*_*m*_ (μM)	*k*_cat_/*K*_*m*_ (M^−1^ s^−1^)
RB19	251 ± 30	138 ± 25	(1.8 ± 0.7) × 10^6^
RB5	144 ± 8	57 ± 7	(2.5 ± 0.5) × 10^6^
DMP	86 ± 2	54 ± 3	(1.6 ± 0.1) × 10^6^
Guaiacol	122 ± 7	45 ± 7	(2.7 ± 0.7) × 10^6^

In the presence of appropriate H_2_O_2_ concentrations, *Il*-DyP4 follows the conventional peroxidase catalytic cycle. Initially, *Il*-DyP4 reacts with H_2_O_2_ to form the high-valent intermediate Compound I* [Fe(IV)–OH Por•…aa] through rapid porphyrin radical migration (equation 5). Compound I* then oxidizes a reducing substrate (typically denoted as RH) by abstracting one electron to generate Compound II (equation 6, E[Fe^IV^=O·porphyrin]), which subsequently undergoes a second one-electron reduction to regenerate the resting enzyme (E[Fe^III^·porphyrin]) and release the product (equation 7) (44). However, under high H_2_O_2_ concentrations (≥50 equivalents in this study), Compound II preferentially reacts with H_2_O_2_ rather than RH, leading to the formation of Compound III (equation 8) (45–47). Compound III formation can result in enzyme inactivation through heme destruction, iron release, or oxidation of amino acid residues (28, 48–50).


(5) $$E[Fe^{III}\cdot porphyrin]+H_{2}O_{2}\to E[Fe^{IV}=O\cdot porphyrin\cdot +]+H_{2}O$$

(6) $$E[Fe^{IV}=O\cdot porphyrin\cdot +]+RH\to E[Fe^{IV}=O.porphyrin]+R\cdot$$

(7) $$E[Fe^{IV}=O\cdot porphyrin]+RH\to E[Fe^{III}\cdot porphyrin]+R\cdot +H_{2}O$$

(8) $$E[Fe^{IV}=O\cdot porphyrin]+H_{2}O_{2}\to Compound\;III+H_{2}O$$

As shown in Table 3, the essentially identical kinetic constants for H_2_O_2_ reduction (*k*_cat_ ~100 s⁻¹; *K*_*m*_ ~80 µM) observed for every substrate demonstrate that the nature of the reductant (anthraquinone or phenol) neither alters the H_2_O_2_ reduction step nor perturbs the canonical catalytic cycle (equation 5 to 7-7) under non-inactivating conditions. Consequently, the divergent activities of *Il*-DyP4 originate from substrate-specific shielding of the enzyme rather than any modification of the core peroxidase mechanism. This unique anthraquinone-directed catalysis against high H_2_O_2_ concentrations exposes a completely new theoretical basis for the pronounced substrate selectivity displayed by DyPs.

**TABLE 3:** Steady-state kinetic constants for the reduction of H_2_O_2_ by *Il*-DyP4 (3.3 nM) with two anthraquinone dyes and two phenols as co-substrates

Substrate	*k*_cat_ (s^−1^)	*K*_*m*_ (μM)	*k*_cat_/*K*_*m*_ (M^−1^ s^−1^)
H_2_O_2_^*a*^ (with 0.1 mM RB19)	100 ± 9	78 ± 17	(1.3 ± 0.3) × 10^6^
H_2_O_2_^*a*^ (with 0.1 mM RB5)	114 ± 10	89 ± 16	(1.3 ± 0.3) × 10^6^
H_2_O_2_^*b*^ (with 1 mM DMP)	113 ± 22	77 ± 24	(1.5 ± 0.6) × 10^6^
H_2_O_2_^*b*^ (with 1 mM guaiacol)	89 ± 9	77 ± 14	(1.2 ± 0.8) × 10^6^

^*a*^ The concentration of H_2_O_2_ for oxidizing anthraquinone substrates is 0.05–1.0 mM.

^*b*^ The concentration of H_2_O_2_ for oxidizing phenolic substrates is 0.01–0.3 mM.

### EPR property analysis of *Il*-DyP4

To further explore the differences in *Il*-DyP4’s response to H_2_O_2_ in the presence of different reducing substrates, we examined the enzyme’s EPR spectra under various conditions. Specifically, we compared the behavior of *Il*-DyP4 in the presence of the anthraquinone dye RB19 and the phenolic compound guaiacol at multiple H_2_O_2_ concentrations.

**Fig 2:**
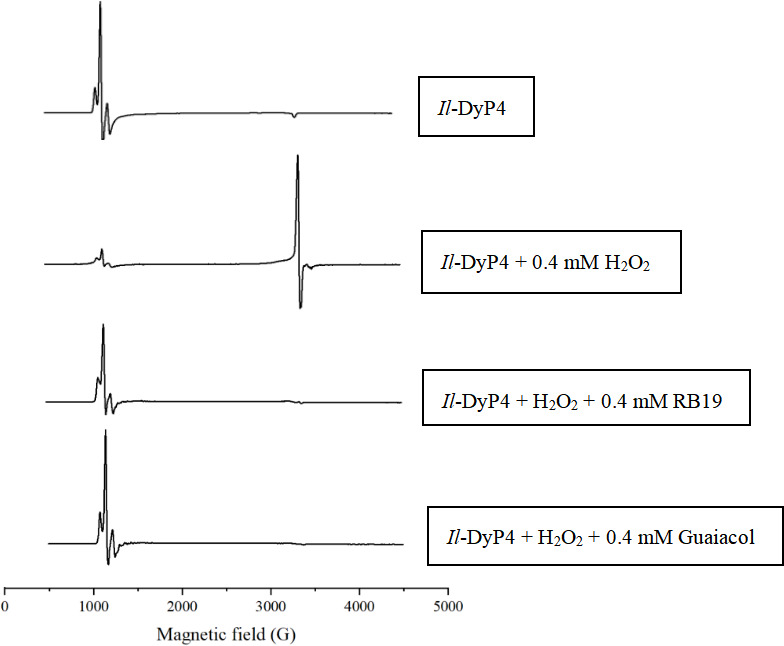
EPR wide-scan spectra of *Il*-DyP4 under low concentrations of H_2_O_2_ (10 eq). All samples (250 μL) contained 40 μM *Il*-DyP4 and 10% glycerol (as a cryoprotectant) in 10 mM sodium tartrate (pH 3.5). The incubation lasts for 20 s. Spectra were collected for the resting enzyme (without substrate or H_2_O_2_), the activated enzyme (containing only H₂O₂), and the complete catalytic cycle of the enzyme (containing 0.4 mM reducing substrate and 0.4 mM H_2_O_2_).

As shown in Fig. 2, resting *Il*-DyP4 exhibited a high-spin heme iron signal centered around 1,000 G. Upon addition of 10 equivalents of H_2_O_2_ (0.4 mM), this signal weakened, accompanied by the emergence of a protein radical signal at *g* ≈ 2.0. This is characteristic of the redox-active amino acid radical formed in Compound I*. EPR spectra of *Il*-DyP4 incubated with either RB19 or guaiacol showed similar changes, consistent with both substrates undergoing oxidation via the canonical catalytic steps (equation 5 and 6), ultimately regenerating the resting enzyme (equation 7). This suggests that both substrate types follow the typical DyP peroxidase mechanism under these conditions.

**Fig 3:**
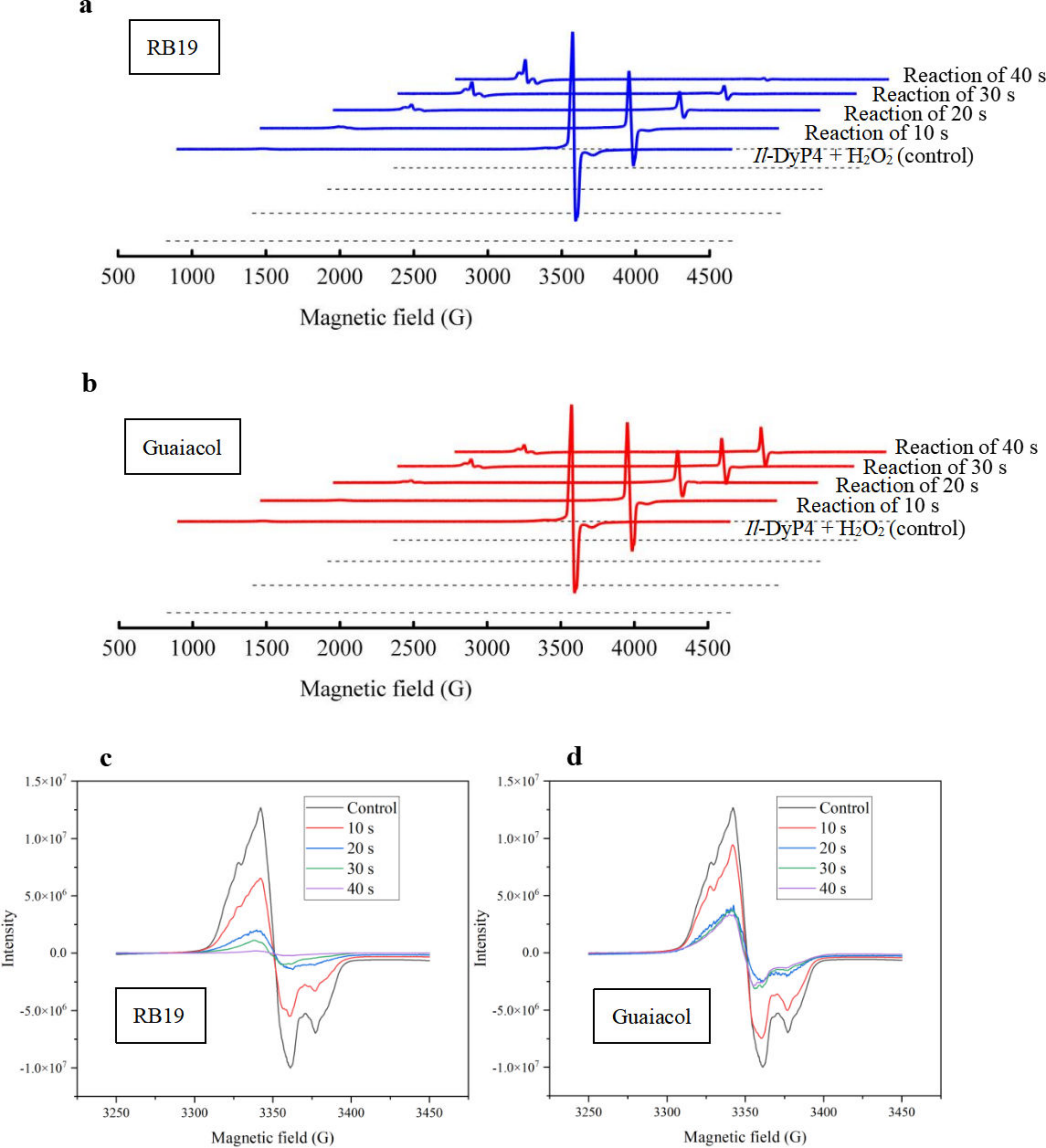
EPR spectra of *Il*-DyP4 recorded over time in the presence of RB19 or guaiacol. (**a and b**) Wide and (**c and d**) narrow scans of reactions with RB19 and guaiacol, respectively. Reaction samples (250 μL) contained 40 μM *Il*-DyP4, 10% glycerol, and 2 mM H_2_O_2_ (50 eq) in 10 mM sodium tartrate (pH 3.5). The radical intensity was measured from 10 s to 40 s after incubation with H_2_O_2_.

To investigate radical dynamics under oxidative stress, we monitored time-dependent radical formation and decay in the presence of 50 equivalents of H_2_O_2_ (2 mM) at time points 0, 10, 20, 30, and 40 s (Fig. 3). In the RB19-containing system, the protein radical signals diminished rapidly, and the high-spin iron signal fully recovered within 40 s, indicating efficient consumption of Compound I* by RB19 (Fig. 3a and c). In contrast, the guaiacol-containing system exhibited a slower and less pronounced decay of radical signals, which plateaued within 20 s (Fig. 3b and d), implying impaired electron transfer and slower catalytic turnover for phenolic substrates. These findings indicate that differences in catalytic efficiency between anthraquinones and phenols primarily stem from the latter two steps of the catalytic cycle (equation 6 and 7). Only anthraquinones effectively complete the full cycle under high H_2_O_2_ conditions by supporting long-range electron transfer that facilitates Compound I* consumption and regeneration of the resting state.

**Fig 4:**
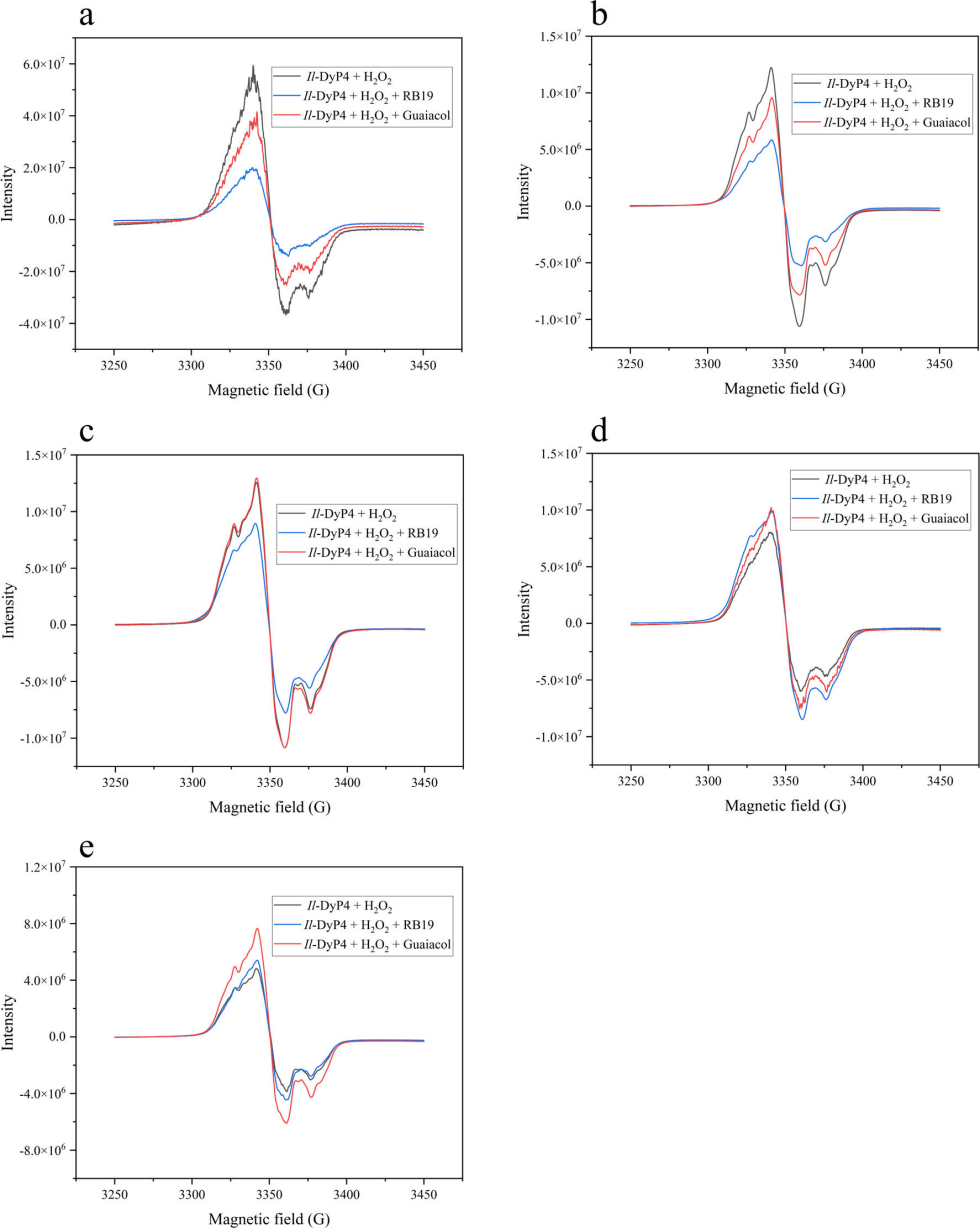
EPR narrow-scan spectra of *Il*-DyP4 with reducing substrates at varying H_2_O_2_ concentrations. Reaction mixtures (250 μL) contained 40 μM *Il*-DyP4, 10% glycerol, and 0.4 mM RB19/guaiacol in 10 mM sodium tartrate (pH 3.5). The incubation lasts for 20 s. (**a–e**) The H_2_O_2_ concentrations were 2 mM (50 eq), 5 mM (125 eq), 10 mM (250 eq), 20 mM (500 eq), and 30 mM (750 eq), respectively.

To further assess this, EPR spectra were recorded after 20 s of reaction at increasing H_2_O_2_ concentrations (2, 5, 10, 20, and 30 mM; Fig. 4). At 2–5 mM H_2_O_2_, radical signals in the RB19 system decayed faster than in the guaiacol system (Fig. 4a and b), reinforcing the superior capacity of RB19 to protect the enzyme by rapidly consuming Cpd I*. At 10 mM H_2_O_2_ (Fig. 4c), the radical signal intensity in the RB19 system fell below that of the control. This decrease indicates that the catalytic cycle remains active: RB19 rapidly reduces the accumulated Compound I radical back to the resting state, thereby preventing the buildup of reactive intermediates. In contrast, the radical intensity in the guaiacol system remained high, similar to the control. This observation aligns with the activity data (Fig. 1), confirming that while the oxidation of guaiacol is inhibited under these high H_2_O_2_ concentrations, the turnover of RB19 proceeds efficiently, protecting the enzyme from accumulation in the radical state. However, at higher H_2_O_2_ concentrations (20–30 mM, 500–750 equivalents), the radical signals in control samples (no substrate) were lower than those in RB19 or guaiacol systems (Fig. 4d and e), indicating that although Compound I* formation still occurs, it is no longer efficiently reduced by either substrate. This aligns with prior observations (Fig. 1d through f), where *Il*-DyP4 lost nearly all activity toward phenolic substrates at ≥250 equivalents of H_2_O_2_. In contrast, significant residual activity (up to ~50%) was maintained toward anthraquinone dyes such as RB19 even at ≥500 equivalents of H_2_O_2_ (Fig. 1a through c), suggesting that RB19 transformation persists beyond the classical peroxidase pathway. These findings imply the involvement of an alternative, possibly non-radical-based mechanism in the anthraquinone transformation process, which cannot be detected by EPR spectroscopy.

### LC–MS analysis of substrate transformation products

**Fig 5:**
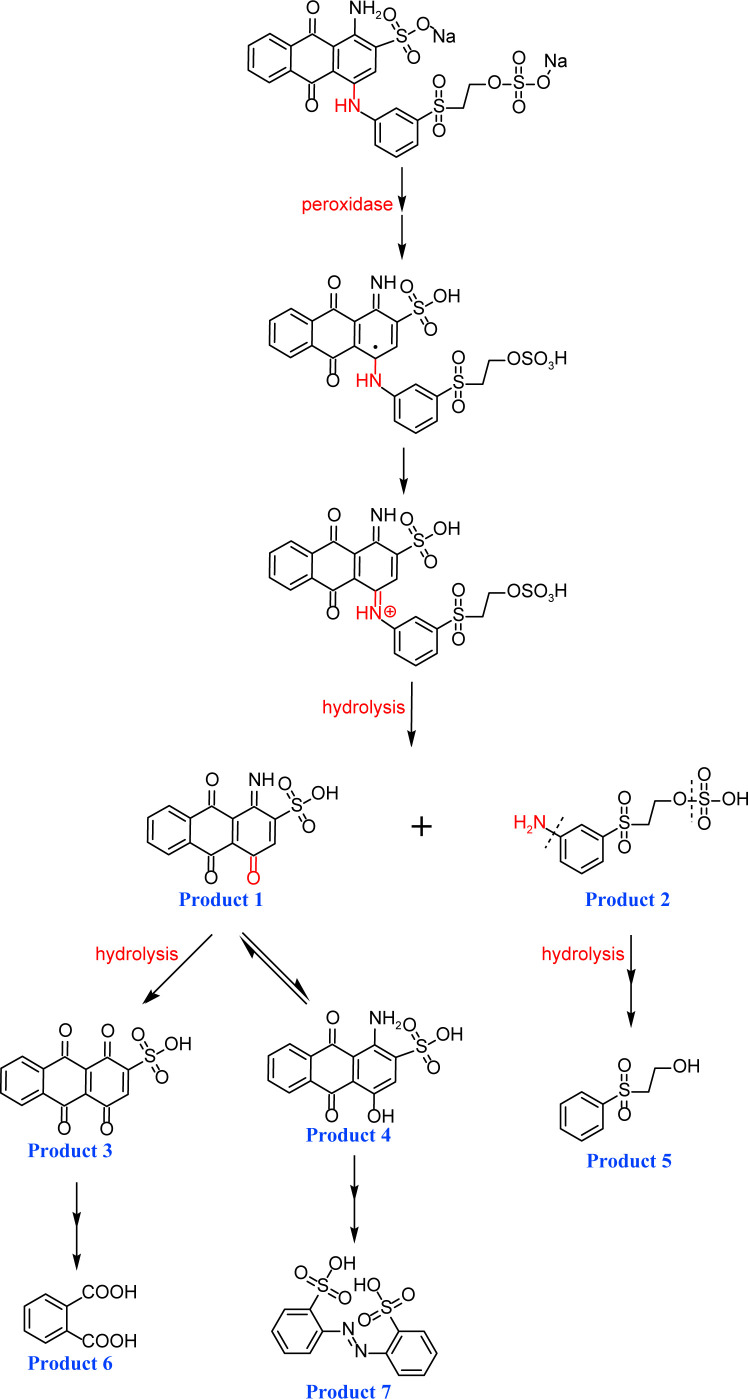
Hypothesized transformation pathway of RB19 by *Il*-DyP4. Reaction mixtures (15 mL) contained 0.1 mM RB19, 0.5 μg/mL enzyme, and 0.1 mM H_2_O_2_ in 10 mM sodium tartrate buffer (pH 3.5). After the reaction proceeded for 5 min at 35℃, freeze-drying was conducted for dehydration, then 1 mL of water was added for redissolution and centrifugation. The resulting supernatant was used for LC–MS analysis.

Based on the LC–MS data obtained in this study, a putative pathway for the *Il*-DyP4-mediated transformation of RB19 was proposed, as illustrated in Fig. 5. Detailed information, including *m*/*z* values, estimated molecular weights, proposed molecular formulas, and structural diagrams of the identified products, is summarized in Table 4 and Fig. S2.

**TABLE 4:** Transformation intermediates of RB19 detected by LC–MS

Product	RB19	Product 1	Product 2	Product 3	Product 4	Product 5	Product 6	Product 7
Molecular formula	C_22_H_16_N_2_Na_2_O_11_S_3_	C_14_H_7_NO_6_S	C_8_H_11_NO_6_S_2_	C_14_H_6_O_7_S	C_14_H_9_NO_6_S	C_8_H_10_O_3_S	C_8_H_6_O_4_	C_12_H_10_N_2_O_6_S_2_
Theoretical exact mass	625.9712	316.9994	281.0028	317.9834	319.0151	186.0351	166.0266	341.9980
Observed *m*/*z*	580.9984	318.0039	279.9948	318.9877	318.0069	185.0240	165.0188	365.9951
Neutral mass	625.9695	316.9966	281.0021	317.9804	319.0142	186.0313	166.0261	341.9986
Proposed structure	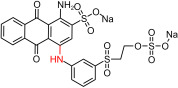	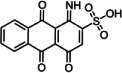	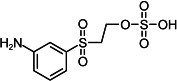	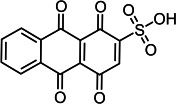	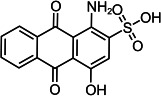	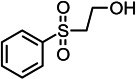	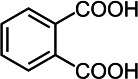	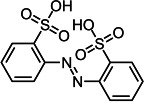

As shown in Fig. 5, the degradation proceeds via a sequential oxidation mechanism consistent with recent mechanistic studies (25, 51). Upon activation by H_2_O_2_, the reactive enzyme intermediates (Compound I* and Compound II) attack the aromatic core of RB19. *Il*-DyP4 initially catalyzes a single-electron oxidation to generate a transient radical intermediate. Subsequently, this unstable radical loses a second electron to yield a cationic imine intermediate (a non-radical species) (51). Crucially, the reaction proceeds in an acidic environment (pH 3.5). Under such conditions, the nitrogen atom of the imine is readily protonated to form a highly reactive iminium ion (C=NH_2_^+^). This protonation creates a strong electron-withdrawing effect, rendering the adjacent carbon atom extremely electrophilic (electron-deficient). Consequently, water molecules, acting as nucleophiles due to the lone electron pairs on their oxygen atoms, rapidly attack this electron-deficient carbon center. This mechanism results in the hydrolytic cleavage of the C=N bond, releasing two stable, non-radical fragments: Product 1 (*m*/*z* 318.0039, C_14_H_7_O_6_NS) and Product 2 (*m*/*z* 279.9948, C_8_H_11_NO_6_S_2_). The cleavage of the C–N bond observed here aligns with the findings of Chaplin et al. (51) and Pi et al. (52). Further analysis of the LC–MS data, in conjunction with the reported degradation products of Reactive Blue 5 by Sugano et al. (50), allowed us to propose a downstream conversion pathway. Specifically, Product 1 is converted successively into Product 3 (*m*/*z* 318.9877, C_14_H_6_O_7_S) and finally to Product 6 (*m*/*z* 165.0188, C_8_H_6_O_4_). In a parallel route, it is transformed into Product 4 (*m*/*z* 318.0069, C_14_H_9_O_6_NS), ultimately leading to the formation of Product 7 (*m*/*z* 365.9951, C_12_H_10_O_8_N_2_S_2_). Both Product 6 (phthalic acid) and Product 7 (2,2′-disulfonyl azobenzene) were identified by ESI-MS and NMR in the earlier study (53). Additionally, Product 2 is hypothesized to be converted into Product 5 (*m*/*z* 185.0240, C_8_H_10_O_3_S) through enzymatic oxidation and hydrolysis. This step is supported by the work of Li et al. (54) on RB19 degradation by lytic polysaccharide monooxygenase and glucose dehydrogenase, in which hydroxyl radical-induced asymmetric cleavage generated an intermediate at *m*/*z* 280, which was further degraded to *m*/*z* 185 via deamination, desulfurization, and oxidation. Through these interconnected steps, RB19 is progressively broken down and ultimately mineralized into small molecular compounds. In summary, the fundamental chemical steps of the RB19 degradation process (e.g., cleavage of the C–NH bond) shown in Fig. 5 are consistent with the mechanisms reported for other DyPs (50–52), but offer new insight by directly linking this specific transformation pathway to the enhanced stability of this enzyme under high oxidative stress.

**Fig 6:**
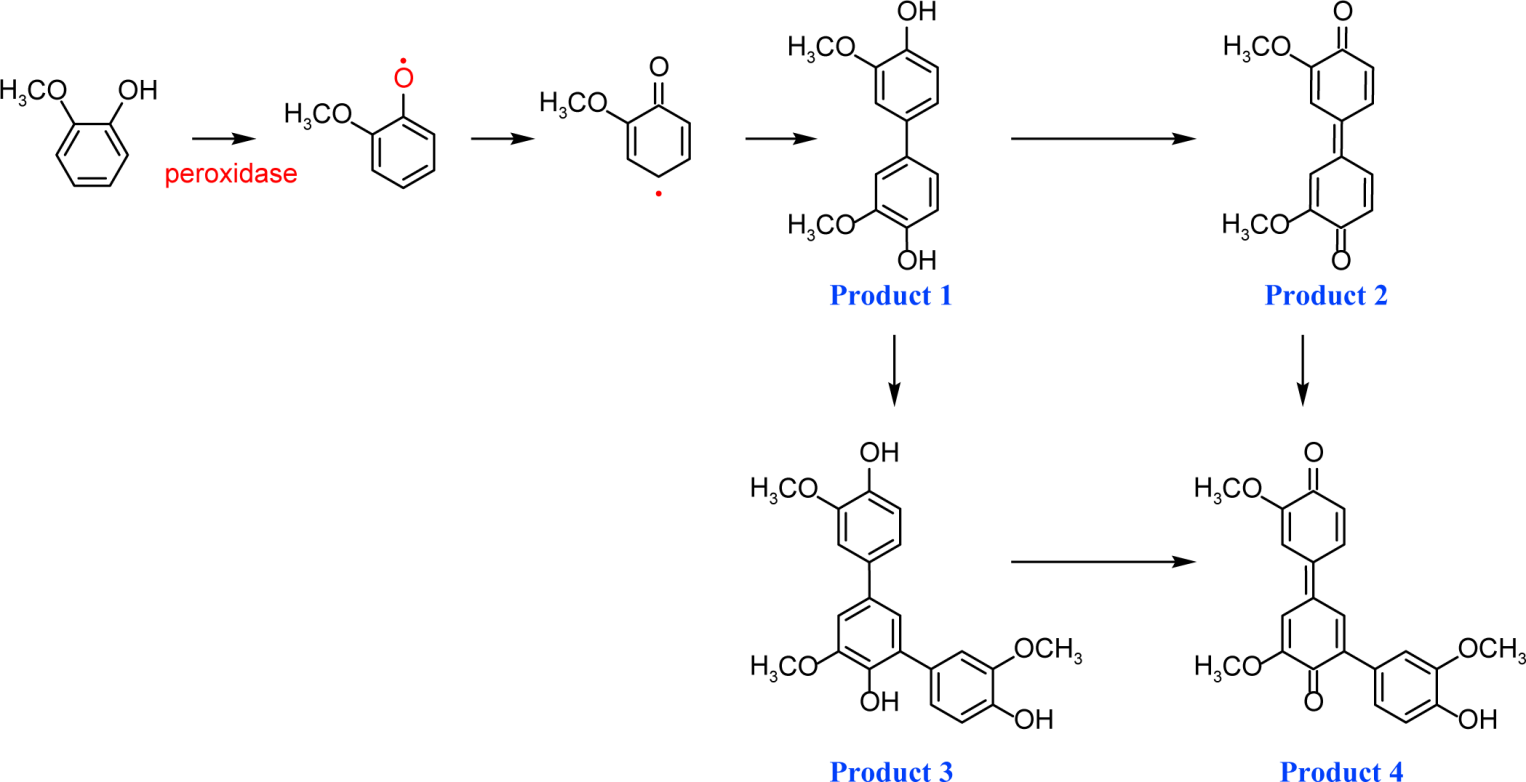
Hypothesized transformation pathway of guaiacol by *Il*-DyP4. Reaction mixtures (15 mL) contained 1 mM guaiacol, 0.5 μg/mL enzyme, and 0.1 mM H_2_O_2_ in 10 mM sodium tartrate buffer (pH 3.5). After the reaction proceeded for 5 min at 35°C, freeze-drying was conducted for dehydration, then 1 mL of water was added for redissolution and centrifugation. The resulting supernatant was used for LC–MS analysis.

By contrast, guaiacol transformation proceeded via classical peroxidase oxidation alone, without any evidence of hydrolytic activity. As shown in Fig. 6, the hydroxyl group of guaiacol is oxidized to a carbonyl, forming a phenoxyl radical that undergoes C–C coupling to produce polymeric products (Products 1–4, the details including *m*/*z* values, estimated molecular weights, proposed molecular formulas, and structural diagrams for them are listed in Table 5 and Fig. S3). Thus, guaiacol transformation by *Il*-DyP4 is restricted to oxidation and subsequent polymerization, with no involvement of hydrolysis. By comparing it with the degradation process of RB19 (Fig. 5), it can be further demonstrated that the hydrolytic cleavage pathway represents an adaptive strategy adopted by *Il*-DyP4 toward RB19. This integrated mechanism, supported by product analysis (LC–MS) and radical kinetics (EPR), explains the unique catalytic preference of DyPs for anthraquinone compounds. To determine whether hydrolysis is a general feature of anthraquinone dye transformation by *Il*-DyP4, we also analyzed intermediates from the transformation of RB5 (the details are listed in Fig. S4 and Table S1). The proposed RB5 transformation pathway shown in Fig. S5 resembles that of RB19, confirming that hydrolysis is a substrate-specific feature of anthraquinone dye transformation by *Il*-DyP4.

**TABLE 5:** Transformation intermediates of guaiacol detected by LC–MS

Product	Product 1	Product 2	Product 3	Product 4
Molecular formula	C_14_H_14_O_4_	C_14_H_12_O_4_	C_21_H_20_O_6_	C_21_H_18_O_6_
Theoretical exact mass	246.0892	244.0736	368.1260	366.1103
Observed *m*/*z*	245.0817	243.0609	367.1180	365.1029
Neutral mass	246.0890	244.0682	368.1253	366.1102
Proposed structure	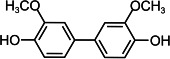	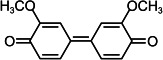	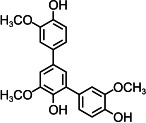	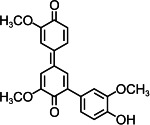

The absolute requirement for both *Il*-DyP4 and H_2_O_2_ for hydrolysis to occur aligns with earlier hypotheses that water derived from H_2_O_2_ activation (37, 55) plays a direct role in facilitating hydrolytic cleavage. Therefore, we propose that *Il*-DyP4 selectively adopts a hydrolytic transformation pathway for anthraquinone dyes, enhancing dye breakdown while reducing H_2_O_2_ accumulation, thereby sustaining the enzymatic activity for efficient degradation. This dual-pathway mechanism provides a compelling explanation for the observed substrate preference and higher catalytic stability of *Il*-DyP4 in the presence of anthraquinone dyes compared to phenolic compounds. Unlike anthraquinones, phenolic substrates do not undergo the specific hydrolytic cleavage that efficiently consumes excess H_2_O_2_ and the radical intermediate. Instead, they rely solely on peroxidase oxidation, which is insufficient to prevent the accumulation of inactivated enzyme species at high H_2_O_2_ concentrations. Consequently, phenolic transformation systems exhibit greater enzyme sensitivity to high H_2_O_2_ concentrations, resulting in rapid inactivation of *Il*-DyP4. To overcome this limitation, Deng et al. (56) proposed alternative activation strategies for generating Cpd I without using H_2_O_2_, which may apply to future enzyme engineering or biocatalytic applications.

### Molecular docking analysis

Previous studies have identified Trp380 (W380) in *Il*-DyP4 as a surface-exposed, radical-forming residue essential for substrate oxidation, including both bulky dyes and small phenolic compounds (19). To further investigate the substrate recognition characteristics of *Il*-DyP4, particularly its affinity for anthraquinones, molecular docking simulations were conducted using RB19 and guaiacol, with a focus on their interaction with the W380 and nearby residues. The predicted binding model between *Il*-DyP4 and RB19/guaiacol is shown in Fig. S6, and the docking results for RB19 and guaiacol with *Il*-DyP4 are summarized in Table 6. The docking scores firstly highlighted a significant difference in binding affinity: RB19 has a score of −6.57 kcal/mol, compared to −3.06 kcal/mol for guaiacol. This indicates that RB19 binds more strongly to *Il*-DyP4 than guaiacol does, which may be attributed to the notable differences in both the types of interactions: RB19 forms two hydrogen bonds (Trp380 and Asn383) and eight hydrophobic interactions (Phe375, Phe386, Phe388, Arg385, Ala379, Gln378, Pro384, and Asn382) with *Il*-DyP4. In contrast, guaiacol engages in two hydrogen bonds (Ala379 and Asn383) and five hydrophobic interactions (Trp380, Phe375, Phe386, Phe388, and Arg385). These results suggested that RB19 relies more heavily on hydrophobic contacts, contributing substantially to binding stability.

**TABLE 6:** Docking results between *Il*-DyP4 and RB19/guaiacol

Ligand	RB19	Guaiacol
Score (kcal/mol)	−6.57	−3.06
Amino acids with hydrogen bond interactions	Trp380, Asn383	Ala379, Asn383
Amino acids with hydrophobic interactions	Phe375, Phe386, Phe388, Arg385, Ala379, Gln378, Pro384, and Asn382	Trp380, Phe375, Phe386, Phe388, and Arg385

In addition, the nature of the interaction between Trp380 and the two substrates differs. RB19 interacts with W380 via hydrogen bonding, whereas guaiacol interacts with W380 through hydrophobic forces. This difference in interaction type is functionally significant: polar interactions, particularly hydrogen bonds, can stabilize enzyme–substrate complexes and enhance catalytic efficiency (43, 57). Therefore, the hydrogen bond between W380 and RB19 likely contributes to the higher binding affinity and catalytic preference of *Il*-DyP4 for anthraquinone dyes over phenolic compounds. These docking results support the biochemical and spectroscopic findings, offering a structural rationale for the observed substrate preference and increased catalytic resilience of *Il*-DyP4 toward anthraquinones under oxidative stress conditions.

Notably, the residue W380 on the surface of *Il*-DyP4 is situated in a hydrophobic environment, surrounded by five hydrophobic phenylalanine residues, as revealed by the high-resolution crystal structure of *Il*-DyP4 (PDB ID: 7D8M) (19). This environment favors the binding of hydrophobic anthraquinone structures but lacks the specific nucleophilic amino acids (such as a Ser-His-Asp catalytic triad) required for enzyme-catalyzed hydrolysis. Furthermore, previous mutagenesis studies confirmed that the W380F mutant almost completely lost its activity toward RB19 (19), which rules out the possibility of potential secondary binding sites being involved in hydrolysis. Consequently, we conclude that the hydrolysis of anthraquinone dyes is a radical-mediated spontaneous chemical reaction. The enzymatically generated cationic imine intermediate undergoes nucleophilic attack by solvent water molecules (pH 3.5). This process is driven by the thermodynamic instability of the intermediate itself, rather than by specific hydrolytic residues within the enzyme.

### Conclusion

DyPs are well known for their substrate preference toward anthraquinone dyes, yet the mechanistic basis for this specificity has remained unclear. This study focused on elucidating the unique catalytic behaviors of the recombinant *Il*-DyP4 in comparison to phenolic compounds, especially under conditions of high concentrations of H_2_O_2_. The transformation pathways for anthraquinone dyes by *Il*-DyP4, encompassing both peroxidase-mediated oxidation and hydrolysis, were proposed. These findings deepen our understanding of DyP catalytic mechanisms and offer a compelling explanation for their substrate preference, thereby advancing the potential application of DyPs in dye-containing wastewater treatment.

## Data Availability

The authors declare that all data supporting the findings of this study are available within the article and its supplemental material or from the corresponding author upon reasonable request.

## SUPPLEMENTAL MATERIAL

Supplemental material aem.00282-26-s0001.docx Figures S1 to S6; Table S1.

ASM does not own the copyrights to Supplemental Material that may be linked to, or accessed through, an article. The authors have granted ASM a non-exclusive, world-wide license to publish the Supplemental Material files. Please contact the corresponding author directly for reuse.
