# Supplementary material for: Unveiling the mechanism of dye-decolorizing peroxidase (DyP): unique anthraquinone-directed catalysis exposes a dual-function peroxidase
Source: Appl Environ Microbiol. 2026 Apr 22;92(5):e00282-26. doi: 10.1128/aem.00282-26 (PMC13188919; doi:10.1128/aem.00282-26)
Supplement: Supplemental material — Figures S1 to S6; Table S1. [file aem.00282-26-s0001.docx]

**Supplementary material for**

**Unveiling the mechanism of dye-decolorizing peroxidase (DyP): unique anthraquinone-directed catalysis exposes a dual-functional peroxidase**

**
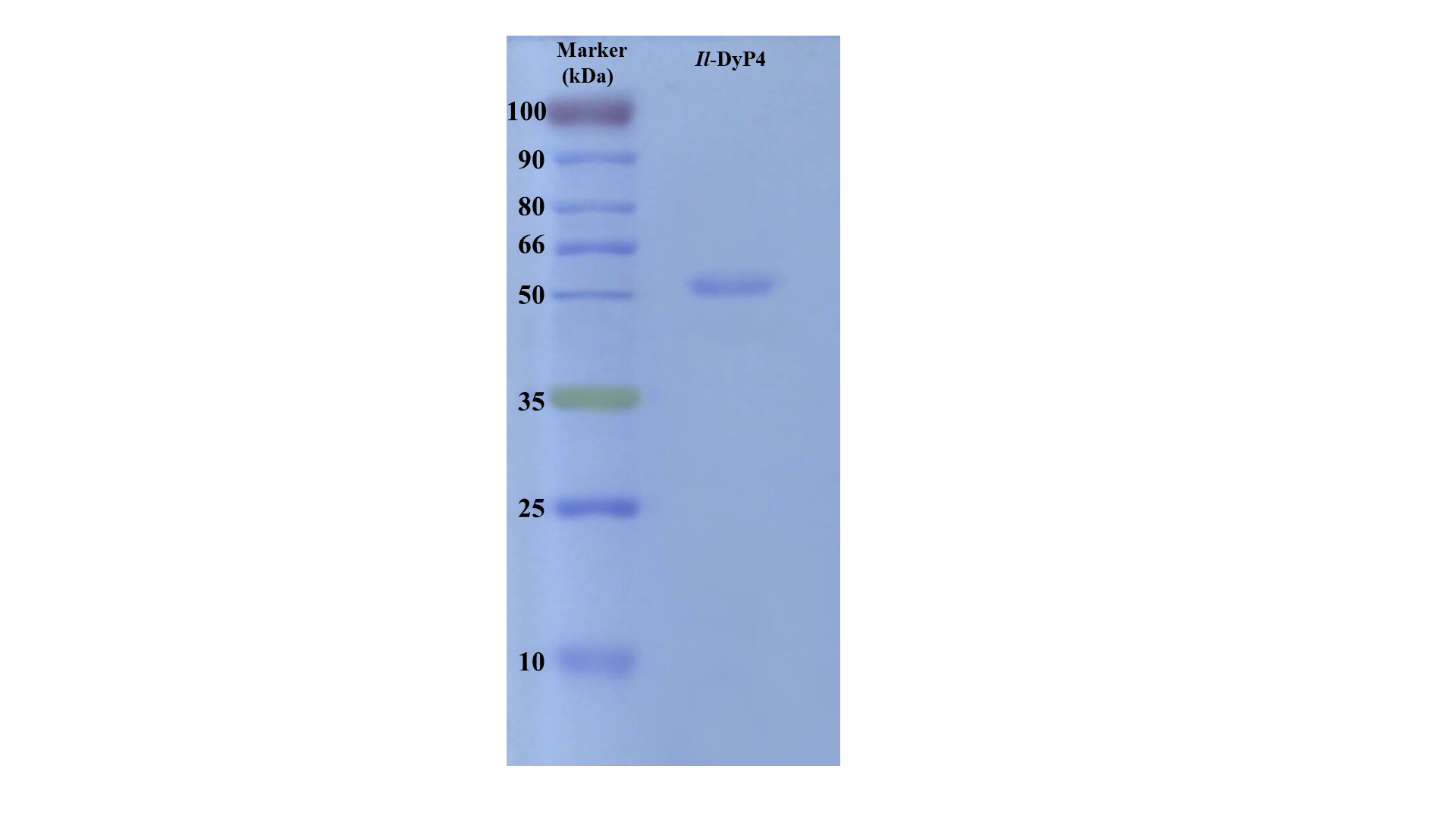
**

*Il*-DyP4 (54 kDa)


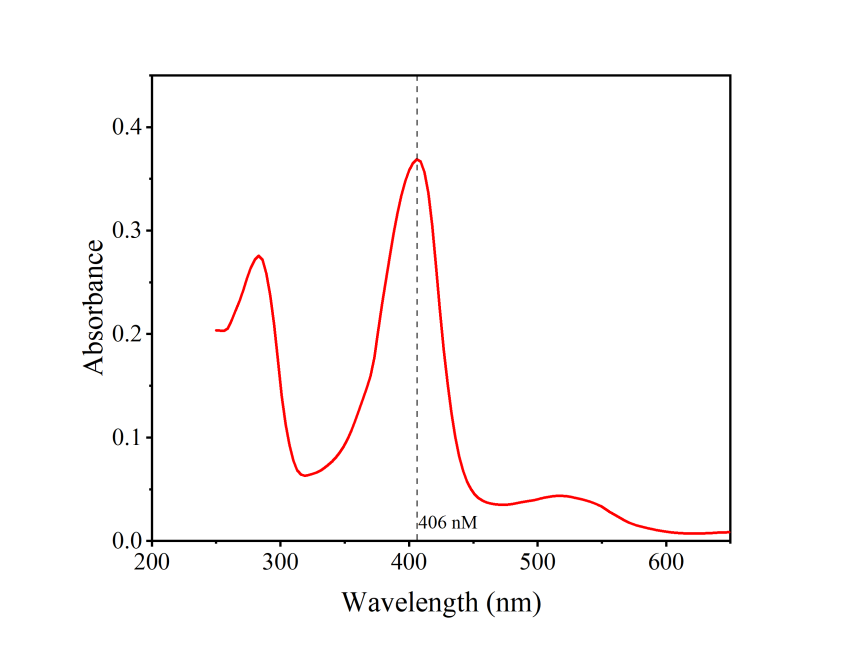


**FIG S1** SDS-PAGE and UV–vis spectra of heterologously expressed *Il*-DyP4.


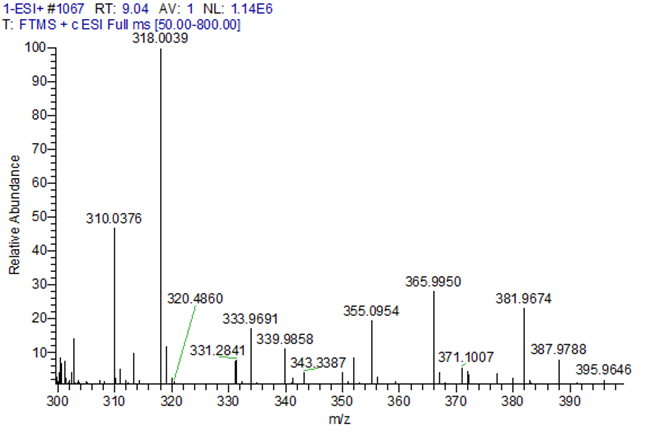


Product 1

1-imino-4,9,10-trioxo-1,4,9,10-tetrahydroanthracene-2-sulfonic acid


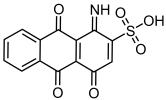

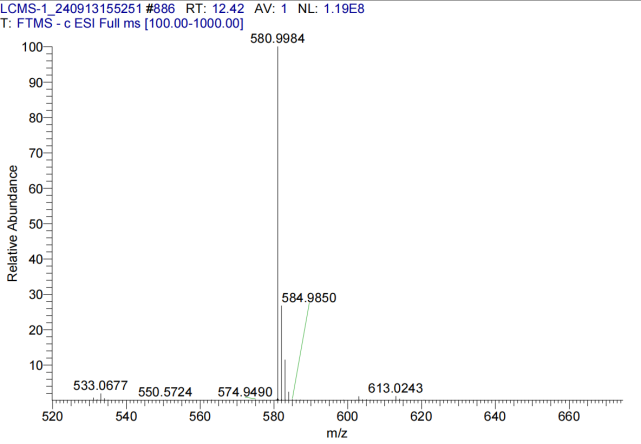


Reactive Blue 19


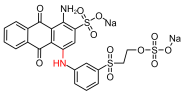

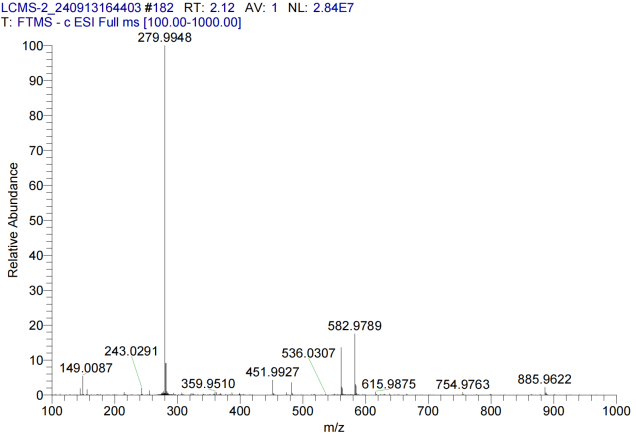


Product 2

2-((3-aminophenyl) sulfonyl) ethyl hydrogen sulfate


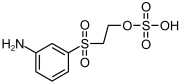

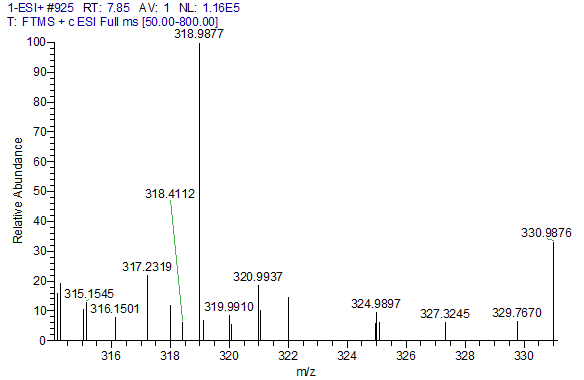


Product 3

1,4,9,10-tetraoxo-1,4,9,10-tetrahydroanthracene-2-sulfonic acid


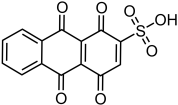


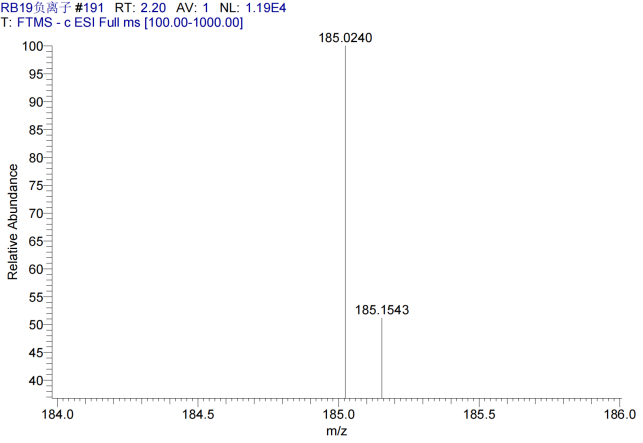


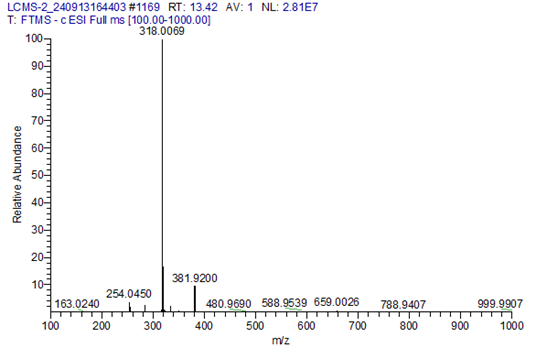


Product 4

1-amino-4-hydroxy-9,10-

dihydroanthracene-2- sulfonic acid


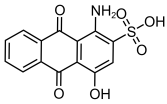


Product 5

2-(Benzenesulfonyl)ethanol


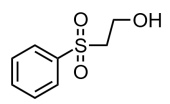

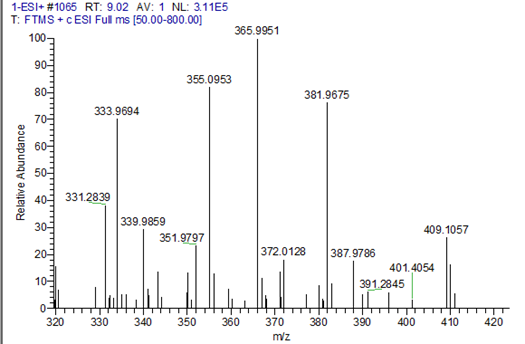


Product 7

2,2’-disulfonyl azobenzene


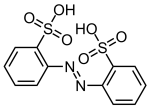

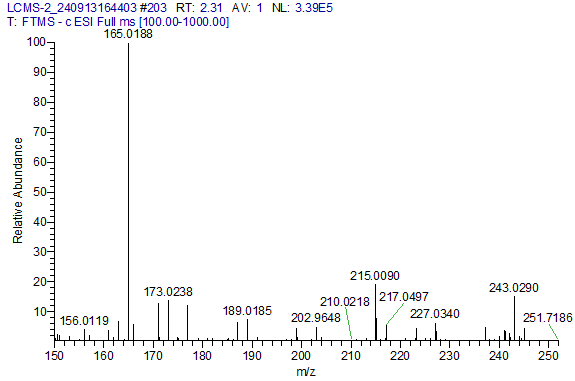


Product 6

Phthalic Acid


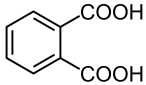


**FIG S2** Mass spectrometry results of RB19 transformation products.


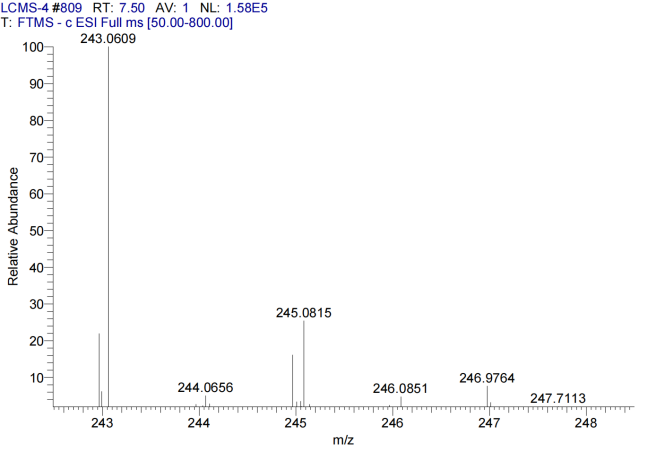


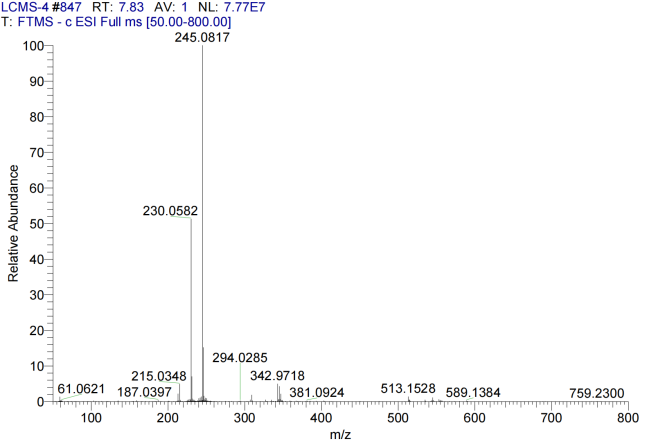


Product 1

3,3'-dimethoxy-[1,1'-biphenyl]-4,4'-diol


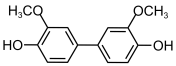


Product 2

3,3’-

dimethoxy-4,4’-biphenoquinone


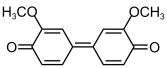

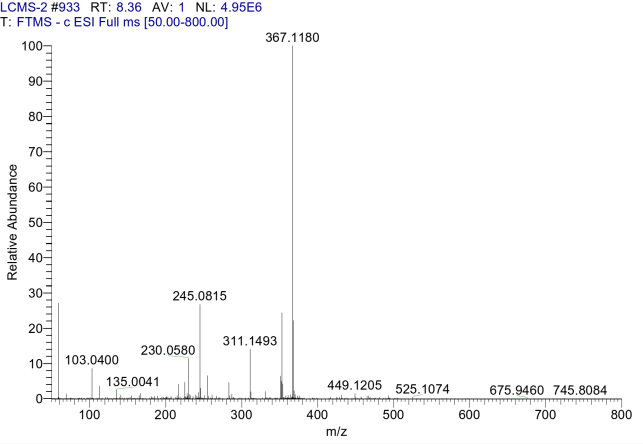


Product 3

3,3'',5'-trimethoxy-[1,1':3',1''-terphenyl]-4,4',4''-triol


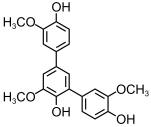

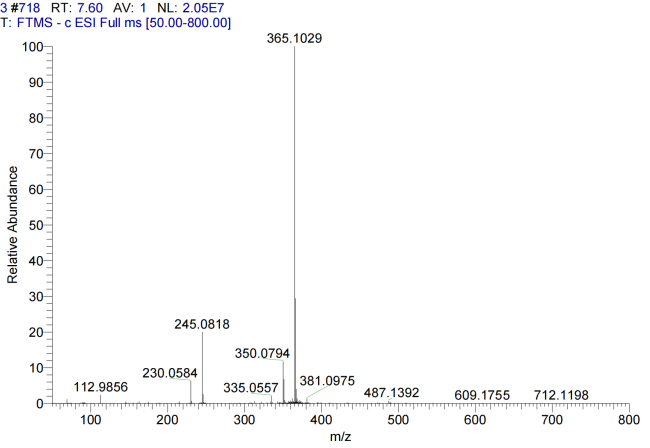


Product 4

(Z)-3-(4-hydroxy-3-methoxyphenyl)-3',5-dimethoxy-[1,1'-bi(cyclohexylidene)]-2,2',5,5'-tetraene-4,4'-dione


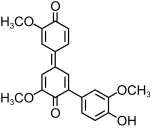


**FIG S3** Mass spectrometry results of guaiacol transformation products.


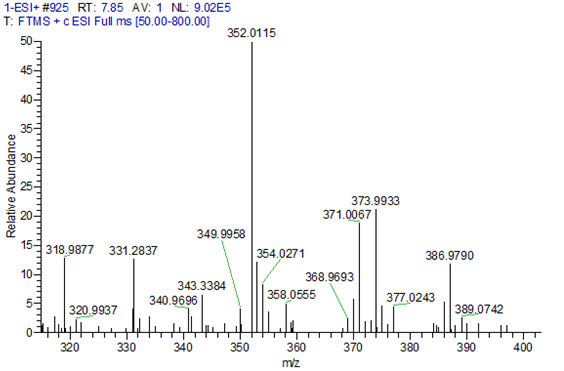


Product 2

1,4,9,10-tetraoxo-1,4,9,10-tetrahydroanthracene-2-sulfonic acid


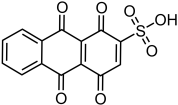

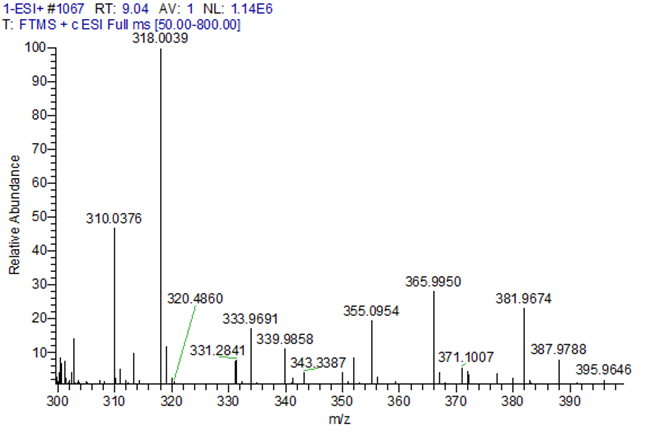


Product 1

1-imino-4,9,10-trioxo-1,4,9,10-tetrahydroanthracene-2-sulfonic acid


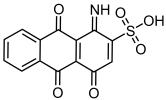

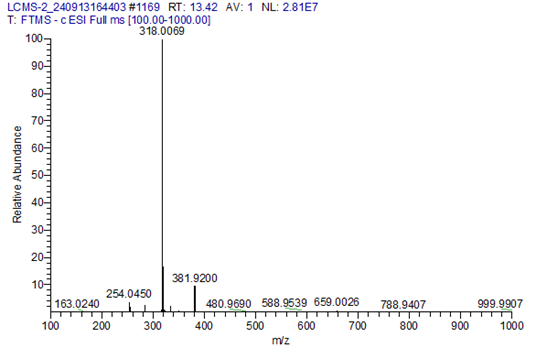


Product 3

1-amino-4-hydroxy-9,10-

dihydroanthracene-2- sulfonic acid


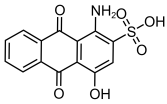

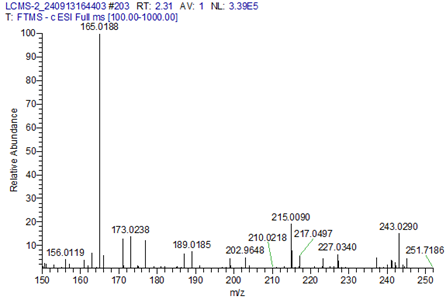


Product 4

Phthalic Acid


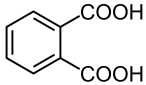

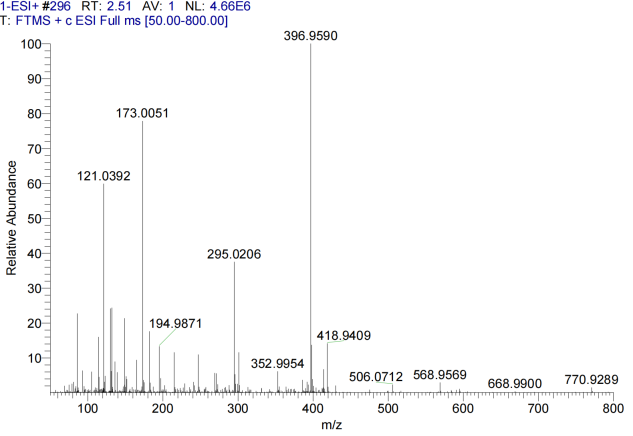


Product 5

2,2’-disulfonyl -4,4’-hydroxy azobenzene


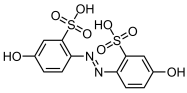


**FIG S4** Mass spectrometry results of RB5 transformation products.


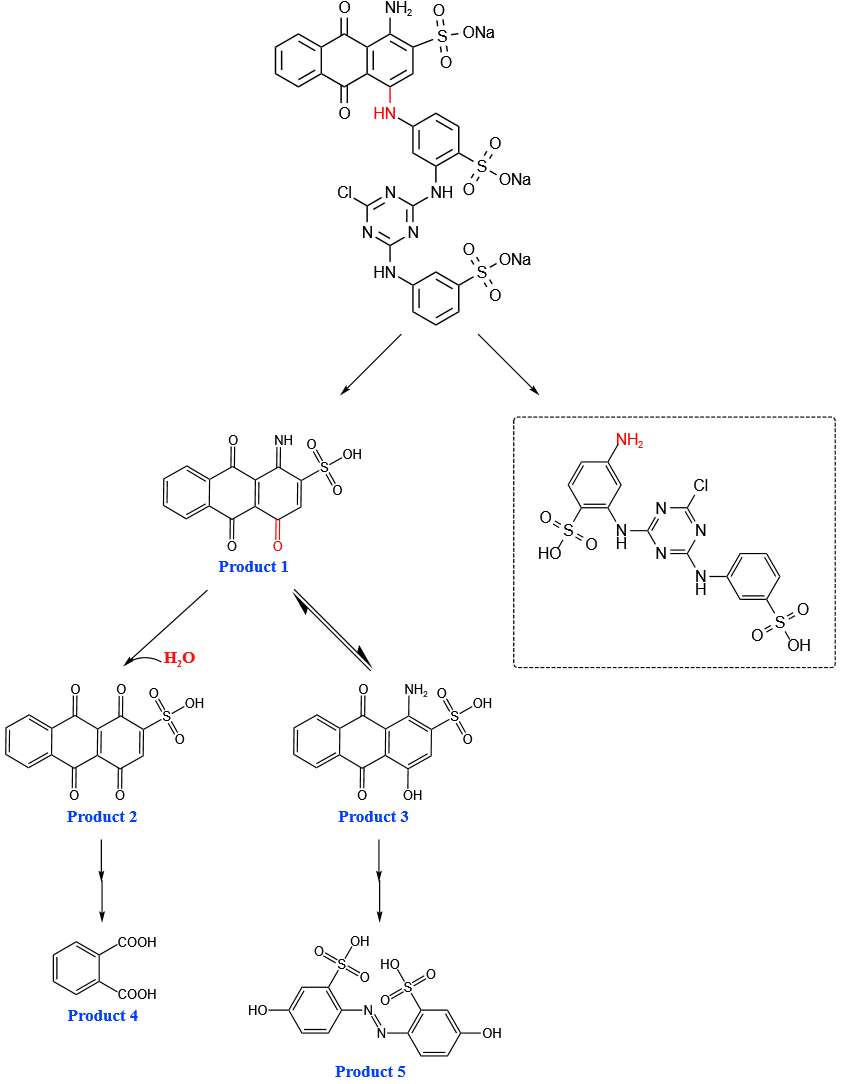


**FIG S5** Hypothesized transformation pathway of RB5 by *Il*-DyP4.

**(b)**

**(a)**


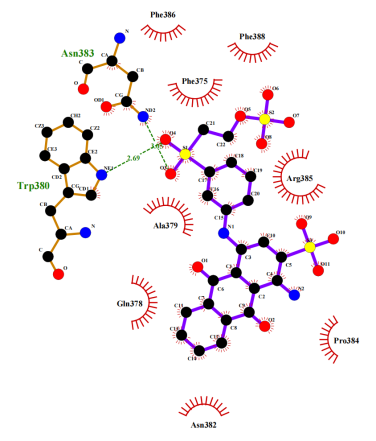

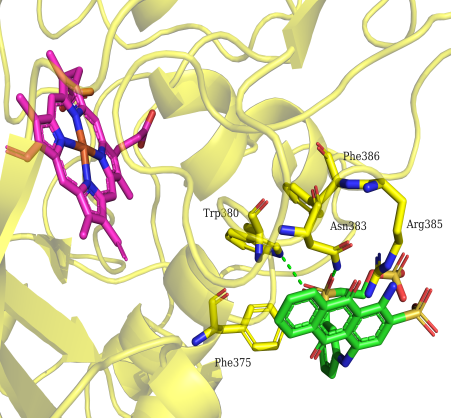


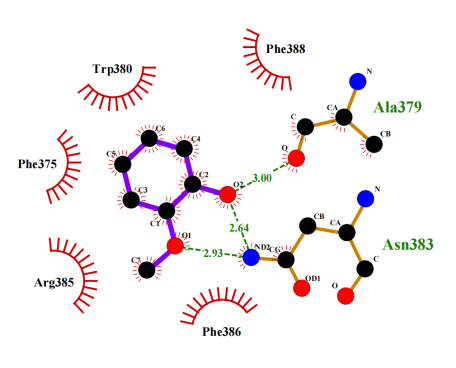

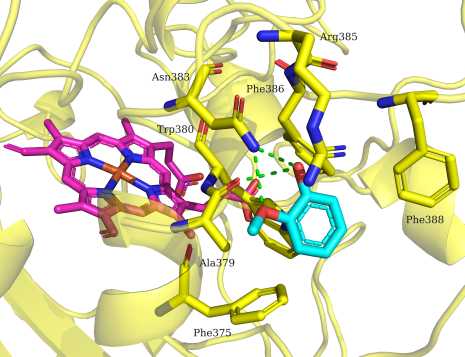


**(d)**

**(c)**

**FIG S6** The predicted binding model between *Il*-DyP4 and RB19/guaiacol. (a, c) The 2D binding mode between *Il*-DyP4 and RB19/guaiacol. (b, d) The 3D binding mode between *Il*-DyP4 and RB19/guaiacol. RB19 was colored green, guaiacol was colored cyan. The heme of *Il*-DyP4 was colored in magenta. The backbone of *Il*-DyP4 was shown as a yellow surface and a cartoon. The residues in the binding pocket of *Il*-DyP4 were shown as yellow sticks. The hydrogen bond interactions were depicted as green dashed lines.

**TABLE S1** Transformation intermediates of RB5 detected by LC-MS

| Product | Product 1 | Product 2 | Product 3 | Product 4 | Product 5 |
| --- | --- | --- | --- | --- | --- |
| Molecular  formulate | C_14_H_7_NO_6_S | C_14_H_6_O_7_S | C_14_H_9_NO_6_S | C_8_H_6_O_4_ | C_12_H_10_O_8_N_2_S_2_ |
| Theoretical exact mass | 316.9994 | 317.9834 | 319.0151 | 166.0266 | 373.9879 |
| Observed m/z | 318.0039 | 318.9877 | 318.0069 | 165.0188 | 396.9590 |
| Neutral mass | 316.9966 | 317.9804 | 319.0142 | 166.0261 | 373.9698 |
| Proposed structure | 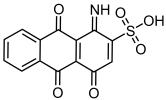 | 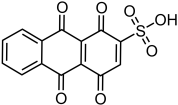 | 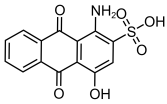 | 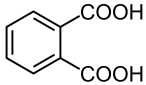 | 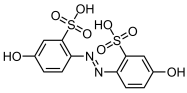 |
